# Role of SCFAs for Fimbrillin-Dependent Biofilm Formation of *Actinomyces oris*

**DOI:** 10.3390/microorganisms6040114

**Published:** 2018-11-13

**Authors:** Itaru Suzuki, Takehiko Shimizu, Hidenobu Senpuku

**Affiliations:** 1 Department of Bacteriology I, National Institute of Infectious Diseases, Shinjuku-ku, Tokyo 162-8640, Japan; itaru122@niid.go.jp; 2 Department of Pediatric Dentistry, Nihon University Graduate School of Dentistry at Matsudo, Chiba 271-8587, Japan; shimizu.takehiko@nihon-u.ac.jp

**Keywords:** biofilm, initial colonization, *Actinomyces oris*, fimbrillin, SCFAs

## Abstract

*Actinomyces oris* expresses type 1 and 2 fimbriae on the cell surface. Type 2 fimbriae mediate co-aggregation and biofilm formation and are composed of the shaft fimbrillin FimA and the tip fimbrillin FimB. Short-chain fatty acids (SCFAs) are metabolic products of oral bacteria, but the effects of exogenous SCFAs on FimA-dependent biofilm formation are poorly understood. We performed two types of biofilm formation assays using *A. oris* MG1 or MG1.Δ*fimA* to observe the effects of SCFAs on FimA-dependent biofilm formation in 96-well and six-well microtiter plates and a flow cell system. SCFAs did not induce six- and 16-hour biofilm formation of *A. oris* MG1 and MG1.Δ*fimA* in saliva-coated 96-well and six-well microtiter plates in which metabolites produced during growth were not excluded. However, 6.25 mM butyric acid and 3.125 mM propionic acid induced FimA-dependent biofilm formation and cell death in a flow cell system in which metabolites produced during growth were excluded. Metabolites produced during growth may lead to disturbing effects of SCFAs on the biofilm formation. The pure effects of SCFAs on biofilm formation were induction of FimA-dependent biofilm formation, but the stress responses from dead cells may regulate its effects. Therefore, SCFA may play a key role in *A. oris* biofilm formation.

## 1. Introduction

The initial attachment of bacteria plays an important role in colonization and interactions with other bacteria on the tooth surface during oral biofilm formation in the human oral cavity [1,2,3,4]. *Actinomyces oris*, formerly called *Actinomyces naeslundii* genospecies 2 [5,6], is a well-known early colonizer that interacts with other bacteria in the oral cavity [6,7]. Fructosyltransferases (FTFs) of *A. naeslundii* synthesize levan, which contributes to cell-cell communication in biofilm formation, but not initial attachment [8]. Levan is an important factor that induces biological activities. *Actinomyces* spp. aggregate with *streptococci* spp. during the progression of dental caries [9] and contribute to periodontal diseases in the presence of levan [9,10,11]. *A. oris* induces co-aggregation with *Streptococcus gordonii* and *Streptococcus sanguinis* as early colonizers and *Fusobacterium nucleatum* as middle colonizers in oral biofilms [1].

*Actinomyces* binds to proline-rich proteins (PRPs) and statherin, which is a phosphate-containing protein in saliva [1]. *A. oris* expresses two types of important fimbriae on its cell surface. Type 1 fimbriae are constructed of shaft fimbrillin FimP and tip fimbrillin FimQ. Type 2 fimbriae are constructed of shaft fimbrillin FimA and tip fimbrillin FimB [12]. Type 1 fimbriae mediate attachment to PRPs on the tooth surface [13], and type 2 fimbriae mediate co-aggregation and biofilm formation [14]. Type 2 fimbriae of *A. oris* are necessary for the recognition of streptococci, which express polysaccharide receptors [15,16].

Short-chain fatty acids (SCFAs) are secreted from numerous oral bacteria, including *Porphyromonas gingivalis* and *Fusobacterium nucleatum* [17,18]. In healthy human volunteers, the concentrations of acetic acid, lactic acid, propionic acid, formic acid, butyric acid and valeric acid were detected in saliva as 6.0 ± 3.5 mM, 1.2 ± 1.9 mM, 1.0 ± 0.8 mM, 0.5 ± 0.5 mM, 0.3 ± 0.4 mM and 0.05 ± 0.2 mM, respectively [19]. High concentrations of butyric acid and propionic acid were mainly detected in periodontal pockets of severe and mild periodontal disease subjects, 2.6 ± 0.4 mM and 0.2 ± 0.04 mM, and 9.5 ± 1.8 mM and 0.8 ± 0.3 mM, respectively [17]. In another report, elevated concentrations of butyric acid (range 4.7–13. 8 mM) and propionic acid (range 87–112 mM) were also detected in dental plaques [20]. Our group reported that 6.25 mM butyric acid increased *A. naeslundii* X600 biofilm in 96-well microtiter plates; SDS-PAGE and Western blotting of biofilm cells demonstrated that the heat shock protein GroEL mediated this upregulation [21]. Another report demonstrated that 60 mM butyric acid increased *A. naeslundii* X600 biofilm in a flow cell system and the initial attachment of *A. naeslundii* X600 cells in six-well culture plates; GroEL also mediated this phenomenon [8]. These reports suggest that butyric acid induces the cell status potential for biofilm formation. However, the relationship between SCFAs and fimbrillin-dependent biofilm formation of *A. oris* is poorly understood.

The present study performed biofilm formation assays to elucidate the relationship between fimbriae and SCFAs. We clearly demonstrated that SCFAs did not induce biofilm formation of *A. oris* MG1 and MG1.Δ*fimA* in human saliva-coated 96-well and six-well microtiter plates. However, butyric acid and propionic acid induced FimA-dependent biofilm formation in a flow cell system. These results in different assay types provide new mechanisms of fimbrillin-dependent biofilm formation stimulated with SCFAs in *A. oris* and oral bacteria producing SCFAs.

## 2. Materials and Methods

### 2.1. Bacterial Strains and Culture Conditions

*Actinomyces oris* MG1 and its derivatives were grown in brain-heart infusion (BHI) broth (BD Diagnostics, Sparks, MD, USA) at 37 °C in a 5% CO_2_ aerobic atmosphere (AnaeroPack; Mitsubishi Gas Chemical Co., Tokyo, Japan). The fimbriae deletion mutant *A. oris* MG1.Δ*fimA* [13,14] was provided by Prof. Hung Ton-That, University of Texas Health Science Center, Houston, Texas. Tryptic soy broth without dextrose (TSB; Difco Laboratories, Detroit, MI, USA) supplemented with 0.25% sucrose (TSBs) with or without different concentrations (3.13, 6.25, 7.5, 10, 15, 20, 30 or 60 mM) of SCFAs (acetic, butyric, formic, lactic, propionic or valeric acids) was prepared for each experiment.

### 2.2. Human Saliva Collection

Human whole saliva samples were collected from 3 healthy volunteers (22~27 years old) after stimulation by chewing paraffin gum and pooled into ice-chilled sterile bottle over a period of 5 min. The samples were clarified by centrifugation at 10,000× *g* for 10 min at 4 °C. Supernatants were transferred into new sterile tubes and sterilized using a 0.22-µm Millex-GP filter (Merck Millipore; Bedford, MA, USA). Sterilized human saliva was stored at −20 °C until use.

### 2.3. Biofilm Formation Assay in 96-Well and 6-Well Plates

Biofilm formation of *A. oris* was assayed using a previously described method [20]. Before the biofilm formation assay, sterilized human saliva was coated on the well from a 96-well culture plate or on a 6-well culture plate sterilized cover glass placed at 4 °C for 1 h. After coating, the wells were washed using sterilized phosphate-buffered saline (PBS), pH 7.4. *A. oris* MG1 and FimA deletion mutants were diluted with flesh TSBs, and bacterial cells were adjusted to an optical density of 0.4 at 600 nm. To analyze the quantity of biofilm formation, twenty suspensions were mixed with 180 μL of TSBs with or without SCFAs, and the mixture was added to human saliva-coated or non-coated 96-well plates and incubated at 37 °C for 6 or 16 h. These concentrations of SCFAs were applied in biofilm formation assay to observe largely the activities of SCFAs. To analyze the quality of biofilm formation, two hundred μL cell suspensions were mixed with 1800 μL of TSBs with or without SCFAs, and the mixture was added to human saliva-coated cover glass in 6-well culture plates and incubated at 37 °C for 16 h. The 96-well or 6-well culture plates were gently washed using PBS, pH 7.4, 2-times and sufficiently dried. Biofilms in 96-well plates were stained with 0.5% safranin for 15 min, washed with distilled water, air-dried, solubilized in a 70% ethanol solution and quantified at an absorbance of 492 nm. The biofilms in 6-well plates were also stained with the FilmTracer *Live/Dead* Biofilm Viability kit (Molecular Probes, Inc., Eugene, OR, USA), which was applied to the biofilms at final concentrations of 5 and 30 µM SYTO 9 and propidium iodide, respectively, and observed using Confocal Laser Scanning Microscope (CLSM). Biofilms were incubated with the dyes at room temperature for 20–40 min and imaged using a confocal microscope (LSM700 Meta NLO CLSM, Carl Zeiss Inc., Thornwood, NY, USA). Confocal images were photographed with a 63× immersion oil objective in the biofilm on the cover glass in the 6-well culture plates. Confocal microscopy acquisition parameters (pinhole, detector and amplifier gain, amplifier offset filters) were set using reference samples and were kept constant in the acquisition of all the remaining images. CLSM images were acquired using an argon laser at 488 and a HeNe-G laser at 555 nm. The independent and triplicate experiments were performed 3 times. Three fields of view were analyzed in each sample. Confocal images of biofilm formation were visually observed using ZEN analysis software (Carl Zeiss, Oberkochen, Germany).

### 2.4. Flow Cell Biofilm Formation Assay

Flow cell biofilm formation of *A. oris* was assayed using the method of Motegi et al [22]. Briefly, *A. oris* MG1 and Δ*fimA* were diluted with flesh TSBs, and bacterial cells were adjusted to an optical density of 0.4 at 600 nm. Cell suspensions were inoculated into chambers of a three-channel flow cell system (Stoval Howcell; Stovall Life Science Inc., Greensboro, NC) and incubated at 37 °C for 3 h. TSBs with or without 6.25 or 60 mM butyric acid and 3.13 or 6.25 mM propionic acid were pumped through the flow cells at a constant rate of 3 mL/h for 48 h using a peristaltic pump (Ismatec; IDEX Corp-Glattbrugg-Zürich, Switzerland). Appropriate concentrations were selected from the results in the 96-well culture plates and from references [8,21] and applied to the flow cell assay. Non-attached cells were removed via washing with distilled water. Confocal images were photographed with a 63× immersion oil objective. Confocal microscopy acquisition parameters (pinhole, detector and amplifier gain, amplifier offset filters) were set using reference samples and were kept constant in the acquisition of all the remaining images. CLSM images were acquired using an argon laser at 488 and a HeNe-G laser at 555 nm. The independent experiments were performed 3 times. Three fields of view were analyzed in each sample. Confocal images of biofilm formation were visually observed using ZEN analysis software. To calculate live and dead cell total areas, the images of the flow cell biofilm cells were re-analyzed with ImageJ 1.48 software.

### 2.5. Statistical Analysis

Comparisons of biofilm levels between two groups were performed using Student’s *t*-tests. Statistical significance was defined at *p*-values < 0.05. Additionally, the comparisons of biofilm levels between multiple groups were by one-way analysis of variance and post-hoc Tukey’s tests. Data were analyzed using Microsoft Excel and SPSS (IBM SPSS statistics 24, IBM Corporation, Armonk, NY, USA).

## 3. Results

**Figure 1:**
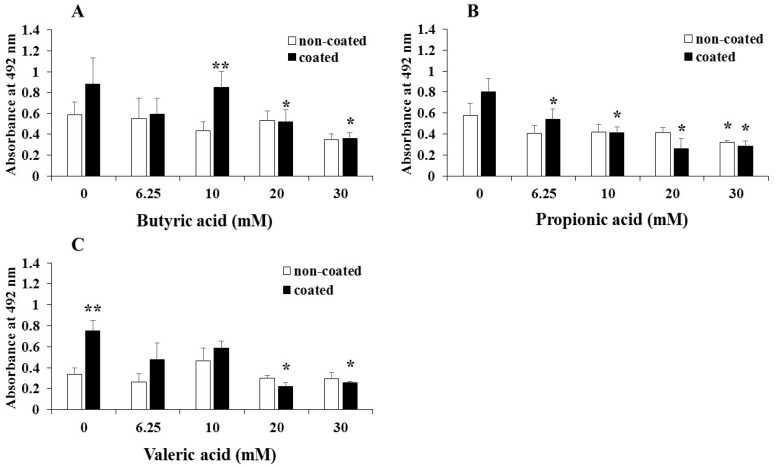
Effect of SCFAs on biofilm formation for six hours in human saliva-coated and non-coated plates. Effects of 6.25–30 mM butyric acid (**A**), propionic acid (**B**) and valeric acid (**C**) on *A. oris* MG1 biofilm formation for six hours in human saliva-coated and non-coated 96-well microtiter plates. The data show the mean ± standard deviation (SD) of three independent assays. The asterisks indicate a significant difference between multiple groups (one-way analysis of variance and post-hoc Turkey’s test: * *p* < 0.05, control: no SCFA vs. concentrations of SCFA) and the two groups (Student’s *t*-test: ** *p* < 0.05, control: no coating vs. saliva-coating).

**Figure 2:**
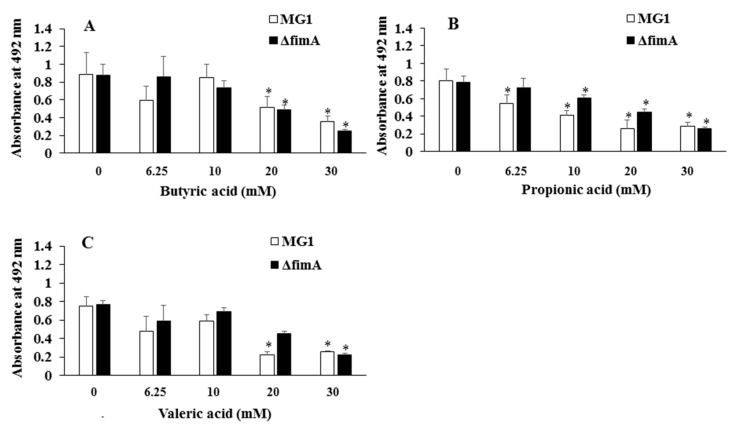
Effects of SCFAs on *A. oris* MG1 and Δ*fimA* biofilm formation for six hours in human saliva-coated plates. Effects of 6.25–30 mM butyric acid (**A**), propionic acid (**B**) and valeric acid (**C**) on *A. oris* MG1 and Δ*fimA* biofilm formation for six hours in human saliva-coated 96-well microtiter plates. The data show the mean ± standard deviation (SD) of three independent assays. The asterisks indicate a significant difference between multiple groups (one-way analysis of variance and post-hoc Turkey’s test: * *p* < 0.05, control: no SCFA vs. concentrations of SCFA).

*Actinomyces* spp. interact with salivary components, such as proline-rich protein and statherin, and form biofilms on tooth surfaces [23,24]. Therefore, the effects of salivary components on six-hour biofilm formation were examined, and the effects of acetic, butyric, formic, lactic, propionic and valeric acids on *A. oris* biofilm formation were observed in human saliva-coated and non-coated plates. Human saliva components enhanced biofilm formation compared to non-coated wells in the absence of SCFAs, 6.25 mM valeric acid and 10 mM butyric acid. However, this was not significant in 0 mM butyric acid, 0 mM propionic acid and 6.25 mM valeric acid (Figure 1). Butyric and valeric acids at 6.25 and 10 mM created equal levels of biofilm formation in the human saliva-coated plates, compared to the no butyric acid control (Figure 1A,C). However, 20 and 30 mM butyric acid, compared to the no butyric acid control, significantly inhibited biofilm formation in the human saliva-coated plate. Propionic acid inhibited biofilm formation in human saliva-coated plates at all concentrations, but not in non-coated wells (Figure 1B). Other SCFAs showed similar data to butyric acid, propionic acid and valeric acid (data not shown). Therefore, we concentrated on the effects of butyric acid, propionic acid and valeric acid on the biofilm formation in the following experiments. The effects of FimA mutation and butyric, propionic and valeric acids on *A. oris* biofilm formation in human saliva-coated plates were examined. Mutation of FimA, compared with wild-type MG1, did not inhibit biofilm formation after six hours in all conditions: no SCFAs, butyric acid, propionic acid and valeric acids (Figure 2).

Propionic acid enhanced Δ*fimA* biofilm formation at 10 mM and 20 mM, and valeric acid enhanced Δ*fimA* biofilm formation at 20 mM compared to the control.

**Figure 3:**
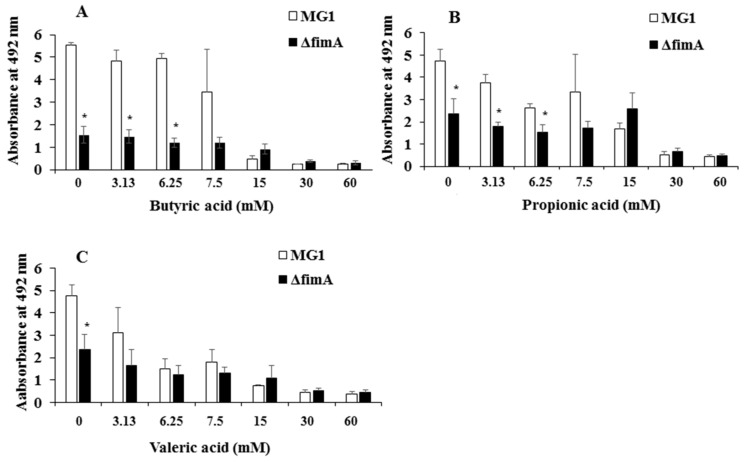
Effects of SCFAs on *A. oris* MG1 and Δ*fimA* biofilm formation for 16 h in human saliva-coated plates. Effects of 3.13–60 mM butyric acid (**A**), propionic acid (**B**) and valeric acid (**C**) on *A. oris* MG1 and Δ*fimA* biofilm formation for 16 h in human saliva-coated 96-well microtiter plates. The data show the mean ± standard deviation (SD) of three independent assays. The asterisks indicate a significant difference between the two groups (Student’s *t*-test: * *p* < 0.05, MG1 vs. Δ*fimA*).

FimA contributes to co-aggregation [25] with streptococci and biofilm formation [26]. Therefore, FimA may play a role during a later stage of biofilm formation. Sixteen-hour biofilm formation assays were observed in conditions with no SCFAs or butyric, propionic and valeric acids in human saliva-coated 96-well microtiter plates to investigate the contribution of FimA at a later stage in biofilm formation. Biofilm formation was significantly lower for MG1.Δ*fimA* than MG1 (Figure 3).

**Figure 4:**
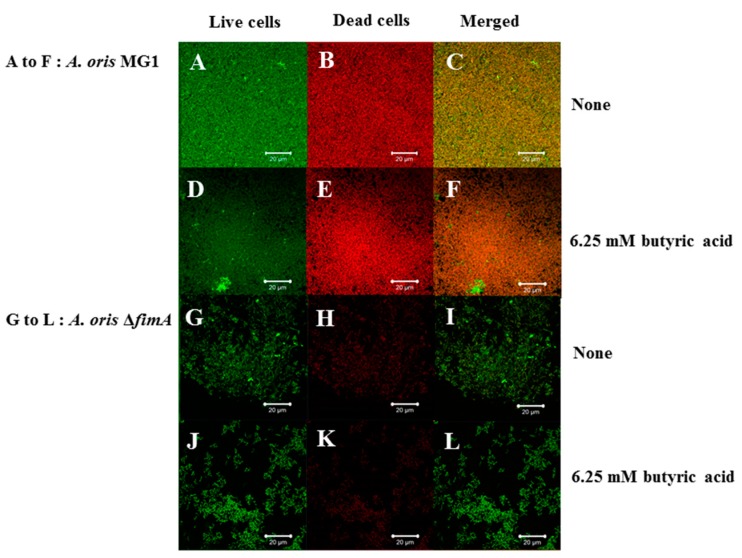
Effects of 6.25 mM butyric acid on *A. oris* MG1 and Δ*fimA* biofilm formation for 16 h on human saliva-coated cover glass in six-well microtiter plates. Effects of 6.25 mM butyric acid on *A. oris* MG1 and Δ*fimA* biofilm formation were observed on human saliva-coated cover glass in six-well microtiter plates. *A. oris* biofilms were stained using a LIVE/DEAD BacLight viability kit and analyzed using a confocal microscope and Zen software. Live and dead cells are indicated with green and red colors, respectively. Live (**left**), dead (**center**) and merged cells (**right**) are present in biofilm stimulated with or without butyric acid. Representative data from more than three independent experiments are presented in the pictures.

**Figure 5:**
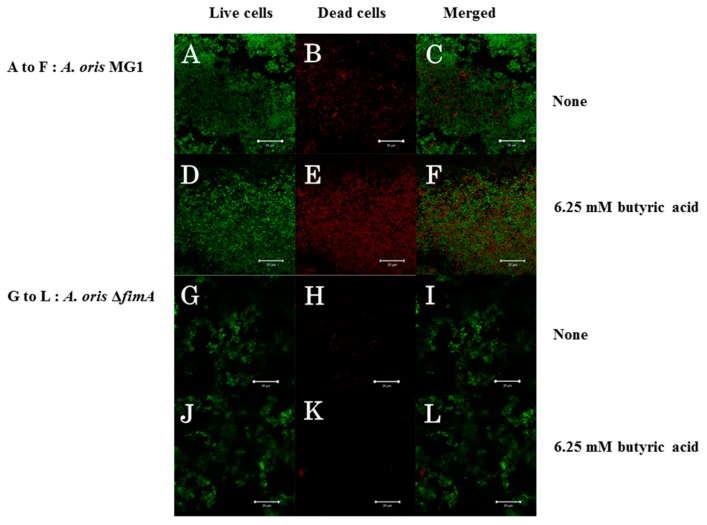
Effects of 6.25 mM butyric acid on *A. oris* MG1 and Δ*fimA* biofilm formation for 48 h in a flow cell system. Effects of 6.25 mM butyric acid on *A. oris* MG1 and Δ*fimA* biofilm formation were observed in a flow cell system. *A. oris* biofilms were stained using a LIVE/DEAD BacLight viability kit and analyzed using a confocal microscope and Zen software. Live and dead cells are indicated with green and red colors, respectively. Live (**left**), dead (**center**) and merged cells (**right**) are presented in biofilms stimulated with or without butyric acid. Representative data from more than three independent experiments are presented in the pictures.

**Figure 6:**
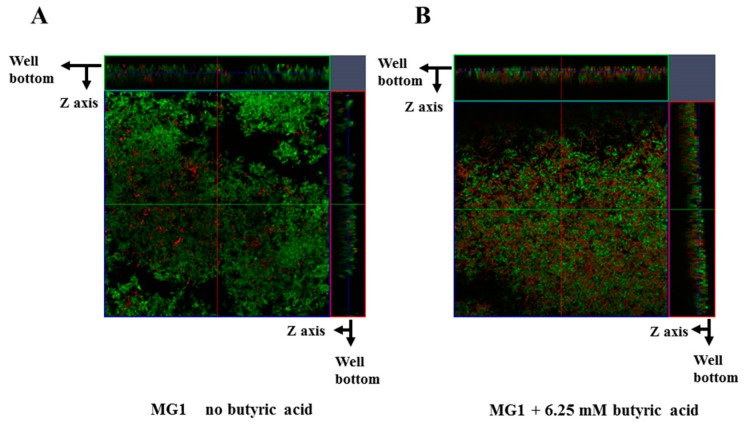
Visual analysis of the effect of butyric acid on biofilm formation of *A. oris* MG1 in a flow cell system. Effects of 6.25 mM butyric acid on the biofilm formation of *A. oris* MG1 were observed and compared with no butyric acid in a flow cell system. The biofilm formation of *A. oris* was stained by a LIVE/DEAD BacLight viability kit and analyzed by confocal microscope and Zen software. Live and dead cells were indicated as green and red color cells, respectively. X-Y and X-Z axis are presented in biofilm stimulated with no butyric acid and butyric acid. Representative data from more than three independent experiments are presented in the picture.

**Figure 7:**
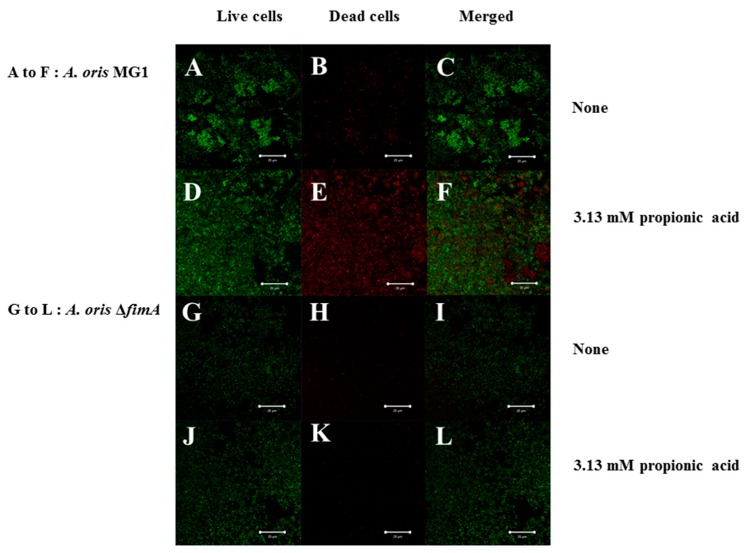
Effects of 3.13 mM propionic acid on *A. oris* MG1 and Δ*fimA* biofilm formation for 48 h in a flow cell system. Effects of 3.13 mM propionic acid on *A. oris* MG1 and Δ*fimA* biofilm formation were observed in a flow cell system. *A. oris* biofilms were stained using a LIVE/DEAD BacLight viability kit and analyzed using a confocal microscope and Zen software. Live and dead cells are indicated with green and red colors, respectively. Live (**left**), dead (**center**) and merged cells (**right**) are presented in biofilms stimulated with or without butyric acid. Representative data from more than three independent experiments are presented in the pictures.

**Figure 8:**
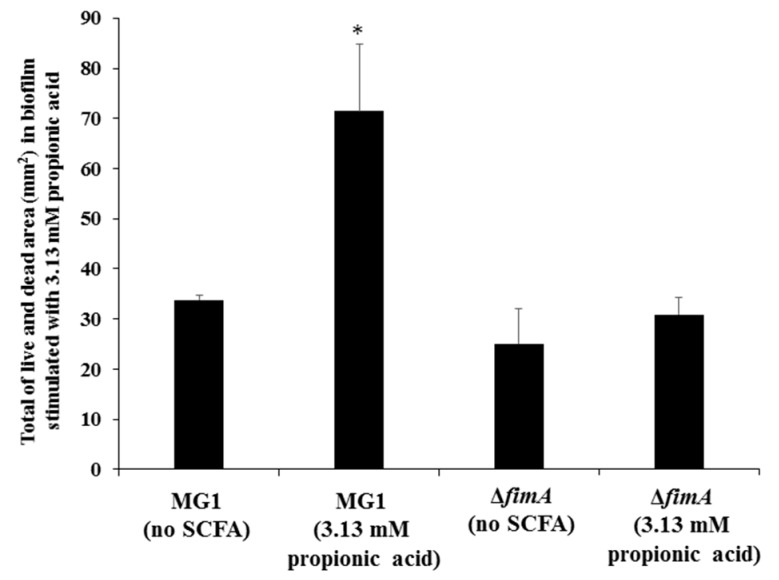
Live and dead cells area (mm^2^) for the effect of propionic acid on biofilm formation of *A. oris* MG1 in a flow cell system. Images from Figure 7 were further analyzed as a mixture of live and dead cell areas (mm^2^) using ImageJ. The data are represented as the mean ± standard deviation (SD) of images from three independent assays. The asterisks indicate a significant difference between the two groups (Student’s *t*-test: *; *p* < 0.05, no SCFA vs. SCFA).

Biofilm formation in conditions with butyric and propionic acids at 3.13 and 6.25 mM was significantly lower for MG1.Δ*fimA* than MG1 mutants (Figure 3A,B). Butyric acid at 3.13 and 6.25 mM induced equal levels of biofilm formation for MG1 and controls with no butyric acid. Propionic and valeric acids inhibited MG1 biofilm formation in a dose-dependent manner beginning at 3.13 mM. All SCFAs slightly inhibited MG1.Δ*fimA* biofilm formation in a dose-dependent manner. Taken together, these results indicate that butyric acid exerted different biological activities from other SCFAs. However, butyric acid, compared with controls without SCFAs, did not enhance *A. oris* biofilm formation in 96-well microtiter plates. The biofilm formation level was similar between 3.13 and 6.25 mM butyric acid and controls without butyric acid (Figure 3). However, the quality of biofilm formation may be different between conditions with and without butyric acid. Therefore, live/dead cell staining was performed for MG1 and MG1.Δ*fimA* biofilm formation in conditions with and without butyric acid (Figure 4). To clearly observe biofilm cells in the culture plate by confocal microscope, the biofilm formation assay was performed on the cover glass coated with human saliva in the six-well culture plates. Live and dead cells were largely observed in MG1 without butyric acid (Figure 4A,B). Merged images revealed that live and dead cells were equally interspersed (Figure 4C). The images of dead cells stimulated with 6.25 mM butyric acid were brighter than images of live cells in biofilm (Figure 4D,E). Merged images indicated that the numbers of dead cells was larger than live cells because an orange color appeared, which indicates that the red staining was mixed more strongly than the green staining (Figure 4F). *A. oris* MG1.Δ*fimA*, compared to MG1, produced a poorer biofilm that included live and dead cells, and 6.25 mM butyric acid did not increase the color of dead cells (Figure 4J–L). Bacteria produce metabolites, including lactic acid, during growth in TSBs. The metabolites mix with SCFAs and may affect *A. oris* biofilm formation*.* To remove the influence of metabolites produced by fermentation during growth, biofilm formation assays were performed using a flow cell system with *A. oris* MG1 and *A. oris* Δ*fimA* in 6.25 mM butyric acid (Figure 5). *A. oris* MG1 formed abundant biofilms in TSBs with and without 6.25 mM butyric acid (Figure 5A–C), but the addition of 6.25 mM butyric clearly increased the number of dead cells compared to the control without butyric acid (Figure 5D–F). The biofilm formation of *A. oris* Δ*fimA* was poor compared to *A. oris* MG1 in TSBs and TSBs with 6.25 mM butyric acid, and 6.25 mM butyric acid did not increase the number of dead cells (Figure 5G–L). Butyric acid (60 mM) did not induce significant biofilm formation (data not shown). To clarify the roles of dead cells in the biofilm stimulated by butyric acid, X-Z axis images are given in Figure 5C,F. Many dead cells attached to the well bottom and largely interacted with live cells in the biofilm induced by butyric acid, but not in the biofilm without butyric acid (Figure 6). Propionic acid (3.125 mM) also promoted dead cells in biofilm formation, but the number of dead cells was fewer than with butyric acid (Figure 7A–L). The biofilm formation of *A. oris* Δ*fimA* was poor compared to *A. oris* MG1 in TSBs with and without 3.13 mM propionic acid, and 3.13 mM propionic acid did not show clear dead cells (Figure 7G–L). Biofilm formation with 6.25 mM propionic acid was similar to that with 3.125 mM propionic acid (data not shown). To clarify the effects of propionic acid on the biofilm formation and the area of attached and aggregated live and dead cells on the flow cells, ImageJ analysis was performed in the images (Figure 7) from the biofilms stimulated with and without 3.13 mM propionic acid. The area of attached and aggregated cells was significantly increased by propionic acid in *A. oris* MG1, but not in *A. oris* MG1.Δ*fimA* (Figure 8). These results demonstrated that butyric acid and propionic acid induced a FimA-dependent biofilm that included more dead cells and altered the characteristics of the biofilm compared to the control biofilm.

## 4. Discussion

*Actinomyces* spp., including *A. naeslundii* and *A. oris*, are among the most abundant microorganisms present in supra- and sub-gingival dental plaques [4,27], and these bacteria play a variety of biological activities in the poly-microbial habitats on the tooth surface [1]. Metabolites, such as SCFAs, from oral biofilm bacteria affect the initial attachment, colonization and biofilm formation of *A. naeslundii* [8,21]. The present study examined the effects of SCFAs on *A. oris* biofilm formation in 96-well microtiter plates coated with human saliva because salivary components interact with *A. oris* [1,2]. Propionic acid and valeric acid inhibited 16-h biofilm formation at all concentrations. However, butyric acid induced levels of biofilm formation at 3.125 and 6.25 mM equal to that of controls and strongly inhibited biofilm formation at concentrations greater than 15 mM. However, more dead cells were observed in the biofilm formation with 6.25 mM butyric acid compared to that of the control. Butyric acid induced dead cell-dependent biofilm formation. This result suggests that damaged cells adhered and aggregated on solid surfaces coated with human salivary components in TSB with butyric acid.

Butyric and propionic acid concentrations are significantly associated with clinical measures of disease severity (e.g., pocket depth and attachment level), inflammation (e.g., subgingival temperature and the percentage of sites bleeding when probed) and total microbial load (all *p* < 0.05) [17]. The attached and colonized bacterial cells in the present experiment were exposed and received biological stimulation, including stresses with 3.13 mM and 6.25 mM butyric acid, which is physiologically observed in periodontal pockets, for 16 h. Our previous report demonstrated that 6.25 mM butyric acid, 3.13 mM propionic acid and 3.13 mM valeric acid upregulated *A. naeslundii* X600 biofilm formation compared to controls without SCFAs in human saliva-coated 96-well microtiter plates [21]. However, these SCFAs did not increase *A. oris* biofilm formation in this study. Therefore, the susceptibilities of *A. oris*, genospecies 2, to SCFAs are different from *A. naeslundii* X600, which belongs in genospecies 1 [28]. *A. oris* is numerically more successful in oral biofilm formation than *A. naeslundii* (as *A. naeslundii* genospecies 1) [29]. *A. oris* exhibited stronger biofilm formation activities than did *A. naeslundii* x600 in human saliva-coated 96-well microtiter plates (Figure 3) [21]. Because the biofilm occupied the full space of wells in conditions without butyric acid, the action of butyric acid to further upregulate biofilm formation is unknown. Therefore, SCFAs may not have upregulated the biofilm in our study because of a physical limitation. Metabolites including lactic acid during cell growth were naturally produced and accumulated in TSBs with SCFAs, and the mixture of metabolites and exogenous SCFAs stresses biofilm cells in 96-well culture plates (Figure 4). Therefore, dead cells might be induced as a stress response in the biofilm, and the mixture of metabolites caused more damages to biofilm cells in 96-well culture plates than in the flow cell system. However, in the flow cell system without metabolite produced during growth, 6.25 mM butyric acid induced more damaged cells than no butyric acid (Figure 5). Extra-cellular DNA (eDNA) is released from dead cells and serves important functions as a factor of attachment to surfaces and an adhesive factor for bacteria during the initial stage of biofilm formation [30,31]. Butyric acid may exhibit biological activities similar to the release of eDNA and lead to biofilm formation.

Type 1 fimbriae mediate binding to *N*-acetyl-beta-d-galactosamine, acidic proline-rich proteins and statherin, and these fimbriae are more common on *A. naeslundii* genospecies 2 (an early plaque colonizer) than genospecies 1 (a late plaque colonizer) [32,33]. Type 2 fimbriae mediate binding to β-linked galactose and galactosamine structures, and these fimbriae are highly prevalent on *A. naeslundii* genospecies 1 and 2. FimA is a type 2 fimbriae that is a very important factor for *A. oris* biofilm formation. FimA did not contribute to 6-h biofilm formation, but largely contributed to 16 h biofilm formation on the human saliva-coated 96-well microtiter plates in the present study (Figure 2 and Figure 3). This result is consistent with the association of FimA with late biofilm development in *A. oris* [34,35,36]. Butyric acid, propionic acid and valeric acid inhibited MG1 and MG1.Δ*fimA* biofilm formation for six and 16 hours in human saliva-coated 96-well microtiter plates. Therefore, the inhibitory effects of high concentrations of SCFAs are likely independent of FimA. A flow cell system was also used to remove the influence of these metabolites during growth and to examine the pure effects of exogenous SCFAs on *A. oris* MG1 and Δ*fimA* biofilm formation. Notably, 6.25 mM butyric acid and 3.13 mM propionic acid increased biofilms that included dead *A. oris* MG1 cells, but did not increase the biofilm of Δ*fimA* in the flow cell system. These data suggest that in in vitro experimental systems, 6.25 mM butyric acid and 3.13 mM propionic acid promote dead cells in FimA-dependent biofilm formation, and in dead cells, cell contents leak out and inherently make the cells stick more to each other, enhancing the appearance of the biofilm. This may link the function of FimA to *A. oris* biofilm formation.

In conclusion, in flow cell systems, 6.25 mM butyric acid and 3.13 mM propionic acid promoted dead cells in FimA-dependent biofilm formation and increased attached and aggregated dead cells. These results indicate that SCFAs from other oral bacteria, such *P. gingivalis* and *F. nucleatum*, stimulated FimA-dependent biofilm formation of *A. oris*, which is involved in stress responses. However, the metabolites produced during cell growth disturbed the SCFA-induced FimA-dependent biofilm formation. Our study did not elucidate the molecules involved in biofilm formation mechanisms. Further studies of the relationship of FimA and SCFAs are necessary to fully understand *A. oris* biofilm formation. To our knowledge, the present study is the first report of an effect of SCFAs on FimA in the in vitro biofilm formation of *A. oris*.

## 5. Conclusions

Metabolites produced during growth disturb biofilm formation of *A. oris* by exogenous SCFAs in human saliva-coated microtiter plates. FimA-dependent biofilm formation that included dead cells was increased by 6.25 mM butyric acid and 3.13 mM propionic acid in flow cell systems. The pure effect of SCFAs on biofilm formation was the induction of dead cells’ attachment to the well bottom and aggregation of dead and live cells in FimA-dependent biofilm formation. Therefore, SCFAs from oral bacteria such as *P. gingivalis* and *F. nucleatum* may play a key role in *A. oris* biofilm formation in the oral cavity.
